# The Relationship between Werner Syndrome and Sinonasal Malignant Melanoma: Two Sibling Cases of Werner Syndrome with Malignant Melanoma

**DOI:** 10.1155/2017/9361612

**Published:** 2017-06-22

**Authors:** Yoshinori Kadowaki, Satoru Kodama, Munehito Moriyama, Masashi Suzuki

**Affiliations:** Department of Otolaryngology, Oita University Faculty of Medicine, 1-1 Idaigaoka, Hazama-cho, Yufu City, Oita 879-5593, Japan

## Abstract

Werner syndrome (WS) is an autosomal recessive disease characterized by premature aging. Malignant tumors such as thyroid carcinoma and malignant melanoma occur frequently in WS patients. We describe 2 siblings with WS who suffered from sinonasal malignant melanoma (MM). Both patients initially experienced nasal obstruction and recurrent nasal bleeding and died within 2 years of the diagnosis of MM. Otolaryngologists should recognize that WS patients have a high risk for head and neck malignant disease, particularly sinonasal MM, even if they are aged below the expected age range and undergo periodic examinations. Furthermore, it is important that WS patients are aware that a prompt nasal examination is indicated if they experience continuous nasal obstruction or recurrent nasal bleeding.

## 1. Introduction

Werner syndrome (WS) is a rare autosomal recessive disease characterized by premature aging [1]. WS is caused by a mutation in the* WRN* gene, encoding RecQ DNA helicase. The mechanism of premature aging remains unknown, and curative treatment has not been developed. Symptoms of WS appear after 10 years of age and include cataract, characteristic dermatological pathology (tight and atrophic skin), characteristic bird-like face, short stature, premature graying of the hair, and thinning of the scalp hair. Currently, the diagnostic criteria for WS can be found in the International Registry of WS [1]. To date, more than 1,200 patients with WS have been reported worldwide, and about 80% of these reported patients are Japanese [2]. In addition to premature aging symptoms, malignant tumors may be associated with WS, including thyroid cancer and malignant melanoma (MM), and a periodic inspection for malignant tumors is recommended in clinical practice for WS patients. Here, we describe 2 siblings with WS and sinonasal MM.

## 2. Case Presentation

A 44-year-old woman, presented with 3 primary MM lesions on the pudendum, left sole, and left heel. She had been diagnosed previously with amenorrhea at the age of 26 years and bilateral cataracts at 37 years. Both her 43-year-old brother and her 37-year-old sister had suffered from bilateral cataracts. The older sister and the brother were suspected to have WS because of the presence of the characteristic bird-like face. The* WRN* protein was not detected in the peripheral blood of any of the 3 siblings, and they were all diagnosed with WS. Their parents were not consanguineous. The older sister underwent surgical removal of the primary MM lesions, followed by chemotherapy with dacarbazine, nimustine, vincristine, and interferon *β*, at the Dermatology Department of our hospital in 2000.

**Figure 1:**
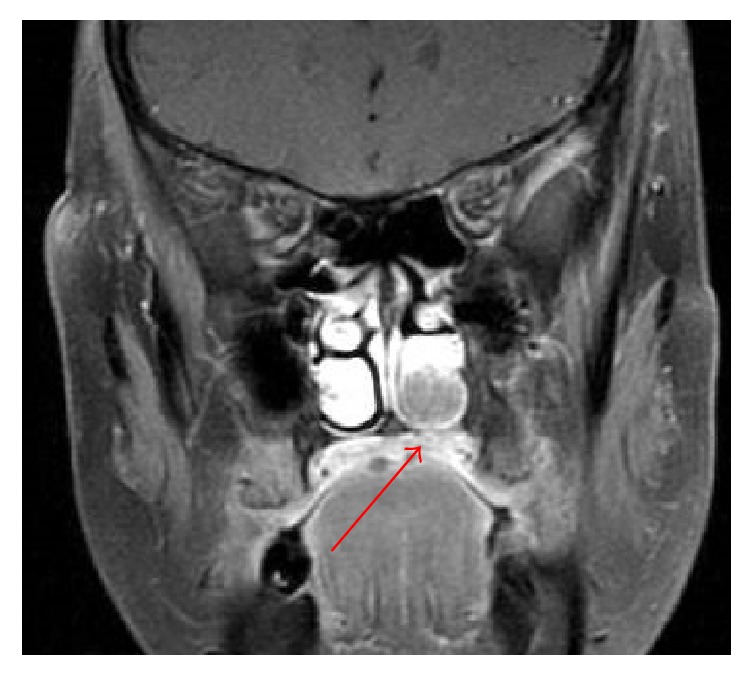
An MRI image shows a tumor (arrow) attached to the left inferior nasal turbinate.

In 2005, the younger sister visited the ENT clinic because she had experienced recurrent nasal bleeding for 1 month. A dark-colored tumor was found in the left nasal cavity. Pathological examination revealed the tumor to be a MM, and the patient was transferred to our department. Magnetic resonance imaging (MRI) revealed a solitary tumor restricted to the left nasal cavity (Figure 1), but an endoscopic examination revealed that a pigmented lesion extended from the tumor and into the nasopharynx. Metastasis to the cervical lymph nodes or other organs was not detected on computed tomography (CT). We staged her as T4bN0M0 according to the AJCC (American Joint Committee on Cancer) classification for cancer of the nasal cavity and paranasal sinuses [3]. She was treated with proton-beam radiotherapy of the nasal cavity. However, left cervical lymph node metastasis was found on MRI within 1 month after the treatment; therefore, she underwent additional proton-beam radiotherapy of her neck and immune-chemotherapy consisting of dacarbazine, nimustine, vincristine, and interferon *β*. Two months later, CT examination revealed metastatic lesions in the liver and sternum. Although surgical treatment and heavy-ion radiotherapy were carried out for each lesion, the metastases continued to expand and spread throughout the whole body. The patient died of metastatic disease 14 months after diagnosis. The dermatologists considered a possible increased risk of sinonasal MM in WS patients; consequently, an annual medical examination at our department was recommended to the other 2 siblings after the younger sister's death to monitor sinonasal MM.

**Figure 2:**
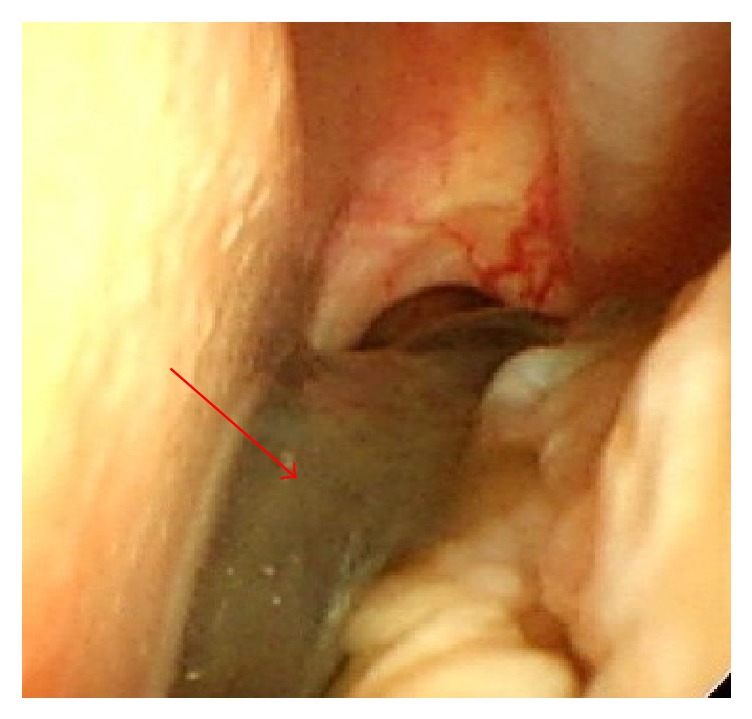
An endoscopic examination image shows a tumor (arrow) in the left nasal cavity.

**Figure 3:**
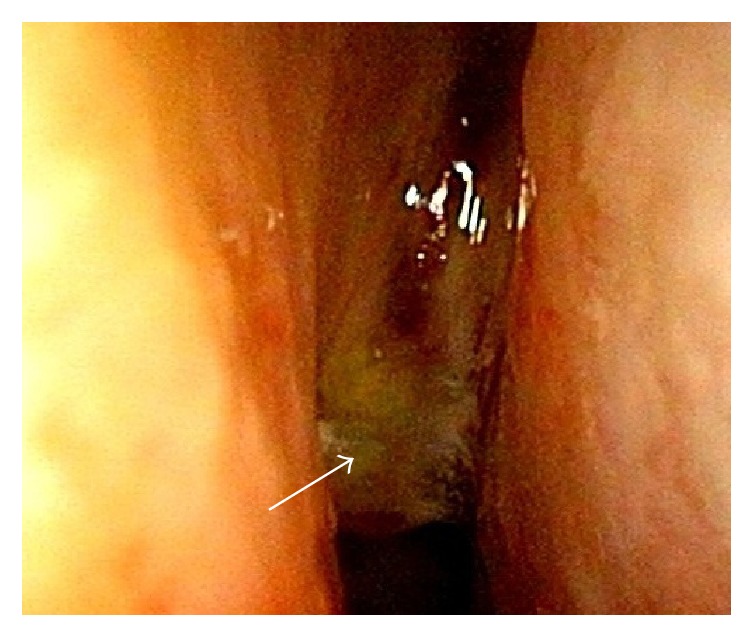
Recurrent MM tumor (arrow) in the right nasal cavity.

**Figure 4:**
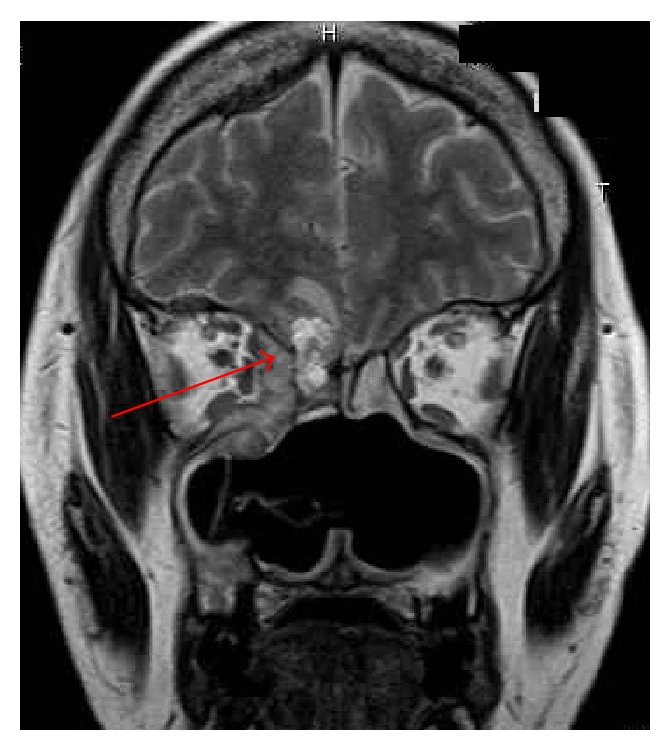
An intracranial invasive lesion (arrow) is shown on this MRI image.

In 2008, the older sister experienced another MM lesion on her right sole, and surgical treatment was performed at the Dermatology Department. In 2010, when she was 54 years old, the value of serum 5-S-cysteinyldopa, a tumor marker for MM, was found to be elevated. Subsequently, she experienced nasal obstruction and recurrent nasal bleeding. Nasal examination showed a dark-colored tumor in the left nasal cavity (Figure 2). Biopsy was performed, and pathological examination revealed that the tumor was an MM. MRI indicated that the tumor originated from the left inferior nasal turbinate and was restricted to the nasal cavity. No metastatic lesion was detected on positron emission tomography-CT (PET-CT). Therefore, we staged the patient as T1N0M0. Endoscopic sinus surgery was selected on the basis of the preoperative findings, and left endoscopic medial maxillectomy was performed under general anesthesia. The tumor was resected with the inferior turbinate and the lateral nasal wall en bloc. Pigmented mucosal lesions were found on the floor of the left nasal cavity and the middle turbinate, and these were excised with safety margins of normal-appearing tissue. The right nasal cavity appeared normal. Because the patient had chronic kidney disease due to the previous chemotherapy, only conventional radiotherapy was applied as postoperative treatment. The patient underwent regular examinations after the treatment, and a new MM lesion was detected in the right nasal cavity 10 months later (Figure 3). CT examination indicated that the tumor was treatable with endoscopic surgery. The patient was initially reluctant to undergo treatment, and endoscopic surgery was only performed 4 months later, at which time the tumor was revealed to be nonexcisable because of intracranial invasion (Figure 4). CyberKnife radiosurgery was performed for the intracranial lesion; however, metastatic lesions were subsequently found in the lumbar vertebrae. The patient underwent palliative care for 2 months and then died of the disease in 2013. The brother has continued to undergo periodic nasal examinations to check for melanoma and has had no appearance of sinonasal MM.

## 3. Discussion

Werner syndrome (WS) is a rare autosomal recessive disease characterized by premature aging, which was first described by Werner in 1904 [4]. WS is caused by a mutation in the* WRN* gene, encoding RecQ DNA helicase. There are heterozygous cases of* WRN* gene mutation with high frequency (1 per 100 people) in Japan [5]; consequently, there is a high prevalence of WS in this country. Symptoms of WS appear after 10 years of age and include cataract, tight and atrophic skin, characteristic bird-like face, short stature, premature graying, and thinning of the scalp hair. The average age of death of WS patients is 55 years, which has been increasing over time, and the main causes of death are cardiovascular disease and malignant disease [6]. WS is known to be accompanied by malignant tumors, including thyroid cancer and MM, and 26.7% of WS patients experienced some type of malignant tumor. The ratio of epithelial to nonepithelial tumors is 1 : 1.9 in WS, as compared with 10 : 1 in the general Japanese population; thus a high prevalence of nonepithelial tumors is observed in WS [7]. The standardized proportionate incidence ratios of thyroid cancer and skin MM in Japanese WS patients compared with the general Japanese population are 9.6 and 59.7, respectively. Furthermore, the mean age of WS patients at first diagnosis of malignant disease is younger than that of the general population [7]. As it is in other parts of the body, the head and neck area is frequently affected by malignant disease in WS patients. Thus, otolaryngologists should examine these patients with attention to this type of malignancy.

MM may occur at wherever melanocytes exist not only in the skin but also in site such as the bulbus oculi, conjunctiva, pharynx, esophagus, sinonasal cavity, and pudendum. Lombardi et al. reported that 0.2–10% of all MMs were mucosal MM and that 50–55% of mucosal MMs were head and neck lesions [8]. On the other hand, Shibuya et al. revealed that 11 of 31 WS patients (35%) with MM had head and neck mucosal MM [9]. Moreover, almost all of the head and neck mucosal MMs (10 cases) were sinonasal MMs. Although sinonasal MM usually occurs in the 6th to 8th decade of life in patient [10], the mean age of the 10 WS patients with sinonasal MM was 47.8 years. Therefore, WS patients may be at high risk for mucosal MM, particularly sinonasal MM, even if they are aged below the expected age range.

We presented 2 siblings with WS who suffered from sinonasal MM. To our knowledge, this is the second report of 2 siblings with WS accompanied by sinonasal MM [11]. Their symptoms included nasal obstruction and epistaxis, which are common in general sinonasal MM patients. In spite of the short period between the appearance of the first symptoms and the start of treatment, both siblings died of the disease within 2 years. Generally, the prognosis of sinonasal MM is known to be poor. Six of the 10 sinonasal MM patients with WS died within 25 months in the report of Shibuya et al. mentioned above; therefore, the prognosis of sinonasal MM in WS patients may be as poor as that in the general population.

Thus, otolaryngologists should recognize that WS patients have a high risk for head and neck malignant disease, particularly sinonasal MM, even if they are aged below the expected range and undergo periodic examinations. Furthermore, it is important that WS patients are aware that a prompt nasal examination is indicated if they experience continuous nasal obstruction or recurrent nasal bleeding.
